# Two-fold elevation of endogenous GDNF levels in mice improves motor coordination without causing side-effects

**DOI:** 10.1038/s41598-018-29988-1

**Published:** 2018-08-08

**Authors:** Kärt Mätlik, Vootele Võikar, Carolina Vilenius, Natalia Kulesskaya, Jaan-Olle Andressoo

**Affiliations:** 1 Department of Pharmacology, Faculty of Medicine & Helsinki Institute of Life Science, University of Helsinki, Helsinki, Finland; 2 Neuroscience Center, Helsinki Institute of Life Science, University of Helsinki, Helsinki, Finland; 3 Institute of Biotechnology, University of Helsinki, Helsinki, Finland; 4 Division of Neurogeriatrics, Department of Neurobiology, Care Sciences and Society (NVS), Karolinska Institutet, Stockholm, Sweden

## Abstract

Glial cell line-derived neurotrophic factor (GDNF) promotes the survival of dopaminergic neurons *in vitro* and *in vivo*. For this reason, GDNF is currently in clinical trials for the treatment of Parkinson’s disease (PD). However, how endogenous GDNF influences dopamine system function and animal behavior is not fully understood. We recently generated GDNF hypermorphic mice that express increased levels of endogenous GDNF from the native locus, resulting in augmented function of the nigrostriatal dopamine system. Specifically, *Gdnf* ^*wt/hyper*^ mice have a mild increase in striatal and midbrain dopamine levels, increased dopamine transporter activity, and 15% increased numbers of midbrain dopamine neurons and striatal dopaminergic varicosities. Since changes in the dopamine system are implicated in several neuropsychiatric diseases, including schizophrenia, attention deficit hyperactivity disorder (ADHD) and depression, and ectopic GDNF delivery associates with side-effects in PD models and clinical trials, we further investigated *Gdnf* ^*wt/hyper*^ mice using 20 behavioral tests. Despite increased dopamine levels, dopamine release and dopamine transporter activity, there were no differences in psychiatric disease related phenotypes. However, compared to controls, male *Gdnf* ^*wt/hyper*^ mice performed better in tests measuring motor function. Therefore, a modest elevation of endogenous GDNF levels improves motor function but does not induce adverse behavioral outcomes.

## Introduction

Ectopic delivery of glial cell line-derived neurotrophic factor (GDNF) protects dopaminergic neurons *in vitro* and in animal models of Parkinson’s disease (PD)^1,2^. Because of this, ectopic GDNF delivery has been tested in a number of clinical trials, one of which is currently ongoing. However, the outcomes have been variable^3–5^, possibly due to differences in methodology and experimental design. In addition to variable efficiency of GDNF treatment in these studies, several side-effects have been reported in both humans and experimental animals. These include massive arborization of dopaminergic fibers towards GDNF injection sites^3^, loss of immunoreactivity of tyrosine hydroxylase – the key enzyme in dopamine synthesis^4^, hyperactivity, and loss of body weight^6–8^, raising the question of whether ectopic GDNF is safe for PD therapy.

We recently generated a mouse model where endogenous GDNF levels are elevated up to 2-fold in the adult brain (GDNF hypermorphs, *Gdnf* ^*wt/hyper*^)^9^. Increase in GDNF expression from the endogenous *Gdnf* locus in *Gdnf* ^*wt/hyper*^ mice was achieved by replacing the *Gdnf* 3′UTR with a 3′UTR less responsive to negative regulation by microRNAs. Because the 3′UTR impacts mRNA stability and translation at the post-transcriptional level, GDNF levels are only increased in cells which naturally transcribe Gdnf. We found that in adult *Gdnf* ^*wt/hyper*^ mice the dopamine system function is altered. More specifically, these mice have ~20% elevated striatal tissue dopamine levels, increased stimulated dopamine release and a 5-fold increase in striatal dopamine transporter (DAT) activity. In addition, the number of dopamine neurons in midbrain substantia nigra and striatal dopamine varicosities are ~15% higher in adult *Gdnf* ^*wt/hyper*^ mice^9^.

Changes in dopamine system function are implicated in several neuropsychiatric diseases, including depression, schizophrenia and attention deficit hyperactivity disorder (ADHD)^10–13^. Furthermore, a reduction in endogenous GDNF levels in *Gdnf* ^*wt/KO*^ mice has been associated with impaired memory and learning^14^, increased anxiety^15^ and enhanced reward value of natural reward sucrose^16^. We thus set out to investigate whether a modest, up to 2-fold ubiquitous increase in endogenous GDNF expression, accompanied by a mild, 15–20% increase in striatal dopamine levels and the number of dopamine synapses, and a substantial increase in striatal DAT activity causes behavioral side-effects. Mice were analyzed with 20 behavioral tests addressing all main central nervous system (CNS) and peripheral functions, including motor coordination, muscle strength, touch sensitivity, nociception, olfaction, locomotor activity, social behavior, sensorimotor gating, anxiety, depression, response to natural reward, impulsivity, and learning and memory. We found that *Gdnf* ^*wt/hyper*^ mice have improved motor coordination compared to the wild-type controls. At the same time, changes in the dopamine system did not cause behavioral deficits associated with dopamine-related neuropsychiatric diseases. These results suggest that 2-fold elevation in endogenous GDNF levels promotes dopamine system function, improves motor function but does not induce adverse behavioral phenotypes.

## Materials and Methods

### Animals

All animal experiments were carried out in accordance with the guidelines laid down in the European Communities Council Directive of 24 November 1986 (86/609/EEC) and were approved by the County Administrative Board of Southern Finland (license numbers ESAVI-2010–09011/Ym-23 and ESAVI/11198/04.10.07/2014).

Altogether 93 male mice (48 *Gdnf* ^*wt/wt*^ and 45 *Gdnf* ^*wt/hyper*^ mice) were tested in four cohorts. In addition, 10 *Gdnf* ^*wt/wt*^ and 9 *Gdnf* ^*wt/hyper*^ female mice were tested in the first cohort. All experiments were performed on gender-matched littermates. A separate batch of female mice (21 *Gdnf* ^*wt/wt*^ and 19 *Gdnf* ^*wt/hyper*^) was tested in the IntelliCage. All mice were maintained in a mixed genetic background^9^.

The mice were group-housed (2–4 animals per cage) with food and water *ad libitum* under a 12-h light-dark cycle (lights on at 6 a.m.) at relative humidity of 50–60% and room temperature 21 ± 1 °C. The bedding (aspen chips, Tapvei Oy, Finland) was changed weekly and a wooden tube and aspen shavings (Tapvei) were provided as enrichment. The age of the mice at the beginning of behavioral testing was 3 months.

### Behavioral tests

The tests were performed in the following order (abbreviations explained in their own paragraphs below) with at least one day in-between subsequent tests:

- Cohort 1 (10 *Gdnf* ^*wt/wt*^ + 10 *Gdnf* ^*wt/hyper*^ males, 10 *Gdnf* ^*wt/wt*^ + 9 *Gdnf* ^*wt/hyper*^ females): OF – LD – HP – RR – BW – GS – PPI – RI – TD – OLF – FC – FST – CLAMS
- Cohort 2 (10 *Gdnf* ^*wt/wt*^ + 9 *Gdnf* ^*wt/hyper*^ males): OF – RR – BW – VG – CH – GS – PPI – FC
- Cohort 3 (13 *Gdnf* ^*wt/wt*^ + 12 *Gdnf* ^*wt/hyper*^ males): OF – RR – BW – VG – CH – GS – PPI – FC
- Cohort 4 (15 *Gdnf* ^*wt/wt*^ + 15 *Gdnf* ^*wt/hyper*^ males): VF
- Cohort 5 (21 *Gdnf* ^*wt/wt*^ + 19 *Gdnf* ^*wt/hyper*^ females): IC

Selection and order of tests was based on a theoretical framework developed for hierarchical behavioral phenotyping with a battery of tests, as well as on relevance to the known functions of GDNF and the dopamine system^17–19^.

#### Spontaneous locomotor activity, open field (OF)

Open field test was used to evaluate general locomotor activity levels and anxiety-like behavior. The mice were released in the corner of a novel open field arena (30 × 30 cm, Med Associates, St. Albans, VT). Horizontal and vertical activity were recorded for 30 minutes at a light intensity of ~150 lx. The peripheral zone was defined as a 6 cm wide corridor along the wall. Distance traveled and the number of rearings were recorded by Activity Monitor v5.1 software (Med Associates, St. Albans, VT).

#### Light-dark exploration (LD)

Light-dark box test was used to assess anxiety-like behavior, as mice have a natural aversion to brightly illuminated areas. The test was carried out in the open field arena (30 × 30 cm, Med Associates, St. Albans, VT) equipped with infrared light sensors detecting horizontal and vertical activity. An opaque insert was used to divide the arena into two halves; an opening (a door with width 5.5 cm and height 7 cm) in the opaque wall allowed animals’ free movement from one compartment to another. Illumination in the center of the light compartment was ~550 lx. The animals were placed in the light compartment and allowed to explore the arena for 10 minutes. Distance traveled, number of rearings, and time spent in different compartments were recorded by Activity Monitor v5.1 software (Med Associates, St. Albans, VT).

#### Rotarod (RR)

Accelerating rotarod (Ugo Basile, Comerio, Italy) test was used to evaluate motor coordination of the mice. The test was performed on two consecutive days. The mice were given three trials a day with an inter-trial interval of 1 hour. Acceleration speed was from 4 to 40 rpm over a 5-min period. The latency to fall off was recorded with the cut-off time set at 6 min.

#### Beam walking (BM)

Beam walking test was used to measure fine motor coordination and balance of the animals. The mice were placed at a point halfway across a horizontal round beam (covered with laboratory tape, outer diameter 2 cm, length 120 cm, divided into 12 sections and raised to 50 cm above floor level). If the mouse fell off within 10 sec, a new trial was started (max. 3 times). The retention time and the number of sections crossed on the beam during 2 min were measured. Results from two trials were averaged.

#### Vertical grid (VG)

Vertical grid test was used to assess motor coordination. The mouse was placed on a horizontal wire grid (25 cm high and 22 cm wide, wire diameter 2 mm spaced 1 cm apart). The grid was raised immediately to a vertical position with the mouse facing the floor at the lower edge. Time taken to turn upward, to climb to the upper edge or to fall off from the grid were measured by stop-watch for a maximum of 1 minute.

#### Coat hanger (CH)

Coat hanger test was used to evaluate forelimb strength and coordination. The animal was placed hanging at a point halfway across of a horizontal bar (diameter 3 mm, length 35 cm) by its forepaws. The body position of the animal was observed for 45 s and was scored as follows:

0 - falls off within 10 sec;

1 - hangs onto the bar by two forepaws;

2 - attempts to climb onto the bar;

3 - hangs onto the bar by two forepaws plus one or both hindpaws;

4 - hangs by all four paws plus tail wrapped around bar;

5 - active escape to the end of bar.

Time to reach the end of the horizontal bar by forepaws (Line 1), to obtain an upward position (forepaws on a diagonal bar at 5 cm from the connection with horizontal part, Line 2) or to fall off was measured.

#### Grip strength (GS)

A commercially available grip strength meter (Ugo Basile, Comerio, Italy) was used to measure forelimb grip strength in mice. The animal was allowed to grasp a bar and was then pulled by its tail. The maximum pulling force (in grams) was recorded when the animal lost its grip on the grasping bar. Five trials were performed with inter-trial intervals of 1–2 min.

#### Tube dominance test (TD)

Tube dominance test was used to evaluate social dominance and aggression. Two unfamiliar mice of the same sex but of different genotypes were placed in the opposite ends of a 30 × 3.8 cm (inner diameter) transparent plastic tube and released simultaneously. The match ended when one mouse completely retreated from the tube. The mouse remaining in the tube was designated the winner and the retreated mouse the loser. Each animal was tested against 5–6 animals. The percent of retreated matches were scored for each animal. Matches lasting more than 2 min or in which animals crossed over each other were not scored. The test was only performed with males.

#### Resident-intruder test (RI)

Resident-intruder test was used to assess aggressive and defensive behaviors. An intruder mouse (unfamiliar sex- and age-matched animal of the same strain) was put in the cage where the test mouse had been habituating for 30 min. Time spent in social activity (sniffing, following, hetero-grooming) and non-social activity (digging, self-grooming, and rearing) was recorded during 5 min observation. The test was only performed with males.

#### Prepulse inhibition (PPI)

Prepulse inhibition was measured to evaluate sensorimotor gating, reflecting the ability of mice to successfully integrate and inhibit sensory information. Mice were enclosed in a transparent plastic tube (inner diameter 4.5 cm, length 8 cm) that was placed and fixed on the platform inside a startle chamber (Med Associates, St. Albans, VT) with a background white noise of 65 dB and left undisturbed for 5 min. Testing was performed in 12 blocks of 5 trials and five trial types were applied. One trial type was a 40-ms, 120-dB white noise acoustic startle stimulus (SS) presented alone. In the remaining four trial types the startle stimulus was preceded by a 20-ms acoustic prepulse stimulus (PPS). The PPS were white noise bursts of 68, 72, 76 and 80 dB. The delay between onset of PPS and SS was 100 ms. The 1st and 12th block consisted of SS-only trials. In remaining blocks the SS and PPS + SS trials were presented in pseudorandomized order such that each trial type was presented once within a block of 5 trials. The inter-trial interval ranged between 10 and 20 s. The startle response, expressed in arbitrary units, was recorded for 65 ms starting with the onset of the startle stimulus. The maximum startle amplitude recorded during the 65-ms sampling window was used as the dependent variable. The startle response was averaged over 10 trials from blocks 2–11 for each trial type. The prepulse inhibition for each PPS was calculated by using the following formula: 100 − [(startle response on PPS + SS trials/startle response on SS trials) × 100].

#### Fear conditioning (FC)

Fear conditioning test was used to evaluate associative learning capabilities of the mice, based on their innate freezing response to fear. The experiments were carried out employing a computer-controlled fear conditioning system (TSE, Bad Homburg, Germany). Training was performed in a transparent acrylic cage (23 × 23 × 35 cm) within a constantly illuminated (~100 lx) fear conditioning box. A loudspeaker provided a constant, white background noise (68 dB) for 120 s followed by a 10 kHz tone [conditioned stimulus (CS), 76 dB, pulsed at 5 Hz] for 30 s. The tone was terminated by a footshock [unconditioned stimulus (US), 0.6 mA, 2 s, constant current] delivered through a stainless steel floor grid (diameter 4 mm, distance 10 mm). Two CS-US pairings were separated by a 30 s pause.

Contextual memory was tested 24 h after the training. The animals were returned to the conditioning box and total freezing time (defined as an absence of any movements for more than 3 s) was measured by infrared light beams scanned continuously with a frequency of 10 Hz. The CS was not used during this time. Memory of the CS (tone) was tested 2 h later in a novel context. The new context was an acrylic box of similar size with black opaque walls and a smooth floor. A layer of wood chips (bedding material) under the floor provided a novel odor to the chamber. After 120 s of free exploration in the novel context the CS was applied for 120 s and freezing was measured as above.

#### Forced swim test (FST)

Forced swimming test was used to assess behavioral despair, a measure of depressive-like behavior. The mouse was placed for 6 minutes in a glass cylinder (diameter 18 cm, height 25 cm) filled with water at 23 ± 1 °C to the height of 15 cm. The animal was judged to be active when struggling, climbing or swimming using all four paws, and immobile when floating passively, being motionless or only doing slight movements with its tail or one hind limb. The time spent immobile was measured in 2 min intervals.

#### Hot plate (HP)

Hot plate test was used to evaluate pain response to heat. A standard hot plate (Ugo Basile) was heated to 52 °C and the mouse was confined there by a Plexiglas cylinder (diameter 19 cm, height 26 cm). The delay before licking or shaking a hind paw was recorded.

#### Automated von Frey (VF)

Touch sensitivity was measured using Dynamic Plantar Aesthesiometer (Ugo Basile). The mice were habituated in the enclosures for 30 min before the start of the test. The probe of the Touch Stimulator was used to apply force on the plantar surface of either of the hind paws. Withdrawal force was measured 3 times from each hind paw and the results were averaged for each animal.

#### Olfactory habituation/dishabituation (OLF)

The olfactory habituation/dishabituation test was used to evaluate whether mice can detect and discriminate between different odors. The mice were placed individually in small clean cages with wood chips on the floor and allowed to adapt for 20 min. Olfactory habituation involved placing the sample (cinnamon or cocoa powder in a perforated Petri dish) in the cage. Time spent sniffing the sample during a 1 min trial was recorded. Habituation was repeated 4 more times with an inter-trial interval of 10 min. To induce olfactory dishabituation, 10 min after the last habituation a novel sample with a different scent was placed in the cage for 1 min and the time spent sniffing was recorded again.

### IntelliCage (IC)

Intellicage test was used to evaluate the animals’ learning and memory, response to natural reward and impulsivity, using different experimental designs described below.

#### Animals

For the IntelliCage, 21 *Gdnf* ^*wt/wt*^ and 19 *Gdnf* ^*wt/hyper*^ female mice at the age of 16–20 weeks at the beginning of experiment were used. One week before the onset of testing, RFID transponders (Planet ID GmbH, Essen, Germany) were injected subcutaneously into the dorso-cervical region under isoflurane inhalation anesthesia. Throughout the experiment the mice were maintained under a 12/12 h light cycle (lights on at 06:00) at controlled temperature (21 ± 1 °C) and humidity (50–60%).

#### Apparatus and procedure

The IntelliCage apparatus (TSE, Germany) was placed in a polycarbonate cage (20.5 cm high, 58 × 40 cm top, 55 × 37.5 cm bottom, Tecniplast, 2000P, Buguggiate, Italy). Its aluminum top contained a freely accessible food rack filled with standard mouse chow (Teklad 2016, Harlan). The floor was covered with bedding (aspen chips 5 × 5 × 1 mm, Tapvei Oy, Finland) and provided 4 central red shelters (Tecniplast, Buguggiate, Italy). Four triangular conditioning chambers (15 × 15 × 21 cm) were fitted in the cage corners and provided room for one mouse at a time. Each chamber contained two drinking bottles, accessible via two round openings (13 mm diameter) which could be closed by motorized doors. Three multicolor LEDs were mounted above each door and the chamber ceiling contained a motorized valve for the delivery of air puffs. Mice that accessed a chamber were identified by a circular RIFD antenna at its entrance (30 mm inner diameter) and the duration of their visit was determined by both the antenna reading and a temperature sensor that detected the presence of the animal inside the corner. During a visit, the number and duration of individual nosepokes at each door were recorded using IR-beam sensors. Licking episodes at each bottle were monitored using lickometers (duration of the episode, number of licks and total contact time were recorded). Recorded data was analyzed with IntelliCage Plus software.

The mice were randomly divided between 3 IntelliCages (13–14 animals per cage) and the following experimental designs were applied.

#### Free adaptation (FA), nosepoke adaptation (NPA) and drinking sessions (DS)

The aim of this experimental design was to habituate the mice to the Intellicage apparatus and a fixed drinking schedule. During the first 6 days in the IntelliCage, all doors were open, providing free access (FA) to all eight drinking bottles. During the next 3 days, all doors were closed but could be opened once per visit with a nose-poke for 5 s (nose-poke adaptation, NPA). During the next 5 days, the mice were adapted to a fixed drinking schedule (DS) with doors opening in response to nose-pokes between the hours of 20:00–22:00 and 04:00–06:00 only.

#### Corner preference (CP), serial reversal (SR), patrolling (PATR) and chaining (CHAIN) tasks

Corner preference, serial reversal, patrolling and chaining tasks were used to evaluate learning and memory of the mice. In this set of tasks, water was available in only one of four corners during each drinking session. The rule predicting the rewarded corner varied between tasks. To begin, water was available in the same corner for 10 sessions (CP), followed by 8 sessions during which the mice had to learn a new corner during each drinking session (serial reversal, SR). To prevent learning by imitation, cage-mates were divided into four sub-groups with different target corners. Next, in the patrolling task (PATR), water was made available in a corner adjacent to the last rewarded corner and the mice were trained for 16 sessions. Finally, the water was always delivered in the corner adjacent to the most recently visited one in which at least one nose-poke had been made, and the mice were again trained for 16 sessions (CHAIN). The direction of patrolling and chaining was assigned randomly for individual mice in the cage (half clockwise and half anti-clockwise). Before switching from SR to PATR and from PATR to CHAIN there was two days of DS where water was available in all corners. Performance in learning tasks was quantified as the percentage of correct visits with nose-pokes.

#### Motor impulsivity (IMP) task

The motor impulsivity task was used to evaluate the ability of the mice to wait for a certain period of time to access water. In this task, all four corners operated in the same way, 24 h per day. The first nose-poke in a visit determined the correct side and initiated a delay period, followed by 5 s during which 3 green LEDs above the door on the correct side switched on and the door opened for drinking. Any nose-poke during the delay period was considered a premature response, whereas the first nose-poke at the open door was counted as a correct response. Correct response latency was defined as the time that elapsed between the onset of the light stimulus and the correct response. The task had three phases. During the first 2 days, delays were set at 0 s (baseline). Then, the delays varied randomly between 0.5, 1.5, 2.5 and 3.5 s for the rest of the task. During the next 2 days, premature responses had no consequence (training). During the final phase of 7 days (testing), premature responses stopped the trial, requiring the mouse to leave the corner and start again.

#### Delay discounting (DD) task

The delay discounting task was used to measure the animals’ response to a natural reward (saccharin). In this task, all four corners operated in the same way, 24 h per day. The task was divided into three phases. First, with delays set at 0 s (i.e. both doors opening upon start of the visit for 7 s) one bottle of water was replaced with 0.5% saccharin in each corner. In two of the corners the bottle containing saccharin was placed on the left side and in the other two on the right side of the corner. The animals were allowed to develop a spontaneous preference for saccharin over 3 days (training). In the second phase (discounting), delays for opening the saccharin doors were increased by 0.5 s every 24 h, whereas the water doors opened as previously without the delay. After 10 days, this resulted in a delay of 5 s. Nose-poke to water during delay time prevented access to saccharin. For the final 3 days (extinction), delays were reset to 0 s. Saccharin preference score was calculated as (number of licks at saccharin bottles/total number of licks) × 100.

### Statistics

Comparisons between two groups were analyzed with Student’s t-test (followed by Welch’s correction, if necessary) or Mann-Whitney U-test. Comparisons between more than two groups were performed with Analysis of Variance (ANOVA) using genotype as an independent variable, or by multiple Student’s t-tests followed by Holm-Sidak correction. ANOVA was completed by within-subjects factors where necessary and appropriate (repeated measurements at different time points, trials or sessions). Post hoc comparisons after significant ANOVA results were carried out by Sidak’s multiple comparisons test. Males and females were analyzed separately. Details and results of statistical analysis are shown in Supplementary Table 1.

### Data availability

The datasets generated and analyzed in the current study are available from the corresponding author on reasonable request.

## Results and Discussion

**Figure 1:**
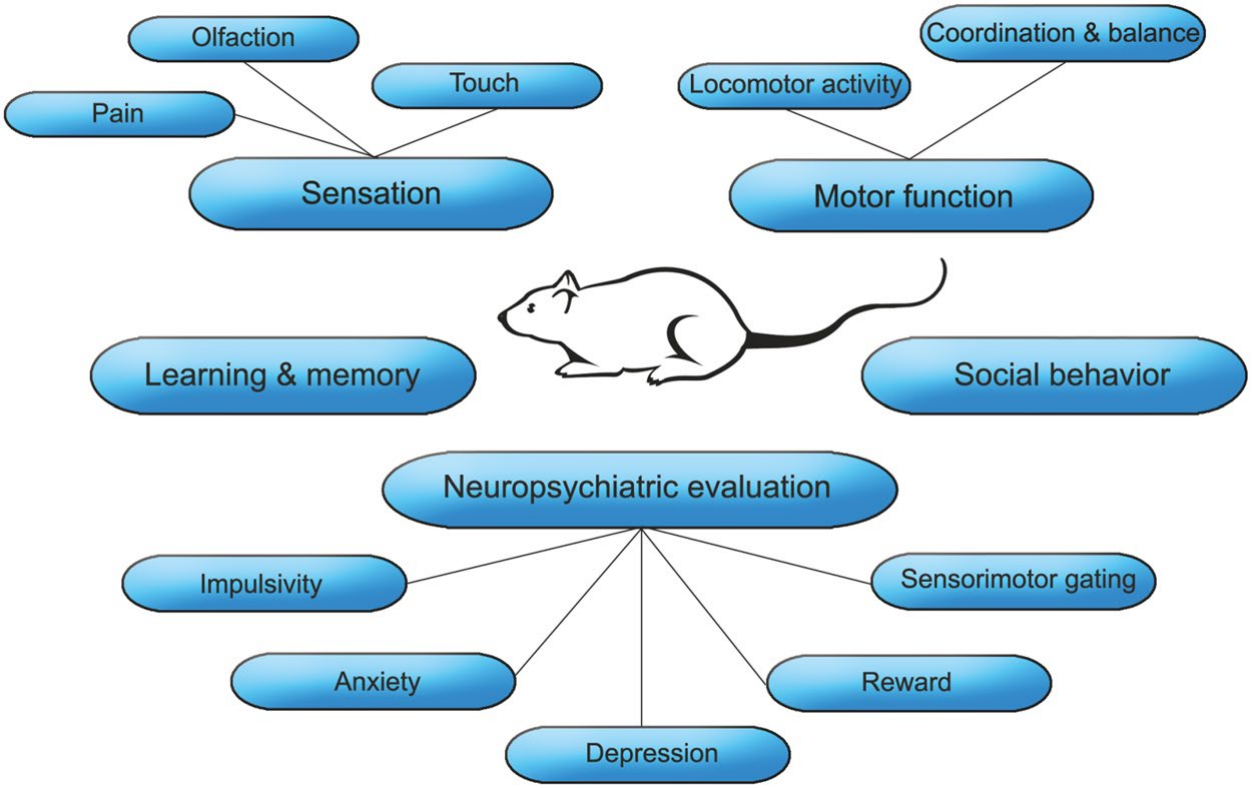
Schematic of behavioral tests used in this study.

An overview of the experiment is shown in Fig. 1 and behavioral tests included in the study are summarized in Table 1. The selected behaviors are associated with dopamine system dysfunction and/or are altered in Parkinson’s disease (PD).

**Table 1:** Behavioral tests included in this study.

Parameter/behavior	Behavioral tests
Motor function	Rotarod (RR)
	Beam walking (BW)
	Vertical grid (VG)
	Coat hanger (CH)
	Grip strength (GS)
Nociception	Hot plate (HP)
	von Frey (VF)
Olfactory function	Olfactory habituation/dishabituation (OLF)
Locomotor activity and anxiety-like behavior	Light-dark exploration (LD)
	Spontaneous locomotor activity (OF)
Sensorimotor gating	Prepulse inhibition (PPI)
Social behavior	Tube dominance test (TD)
	Resident-intruder test (RI)
Depression-like behavior	Forced swim test (FST)
Natural reward	IntelliCage – saccharin preference (SP) and delay discounting (DD) task
Impulsivity	IntelliCage – motor impulsivity (IMP) task
Learning and memory	Fear conditioning (FC)
	IntelliCage – drinking sessions (DS), corner preference (CP), serial reversal (SR), patrolling (PATR) and chaining (CHAIN) tasks

### Motor function

Development of motor symptoms is the hallmark of PD and forms the basis for clinical diagnosis. *Gdnf* ^*wt/hyper*^ mice are protected in a PD mouse model^9^ and GDNF is expressed in a number of brain regions and tissues involved in motor coordination and learning, including the striatum, cerebellum, spinal cord and muscle^20–23^. Therefore we were interested in analyzing the performance of *Gdnf* ^*wt/hyper*^ mice in tests measuring motor function.

**Figure 2:**
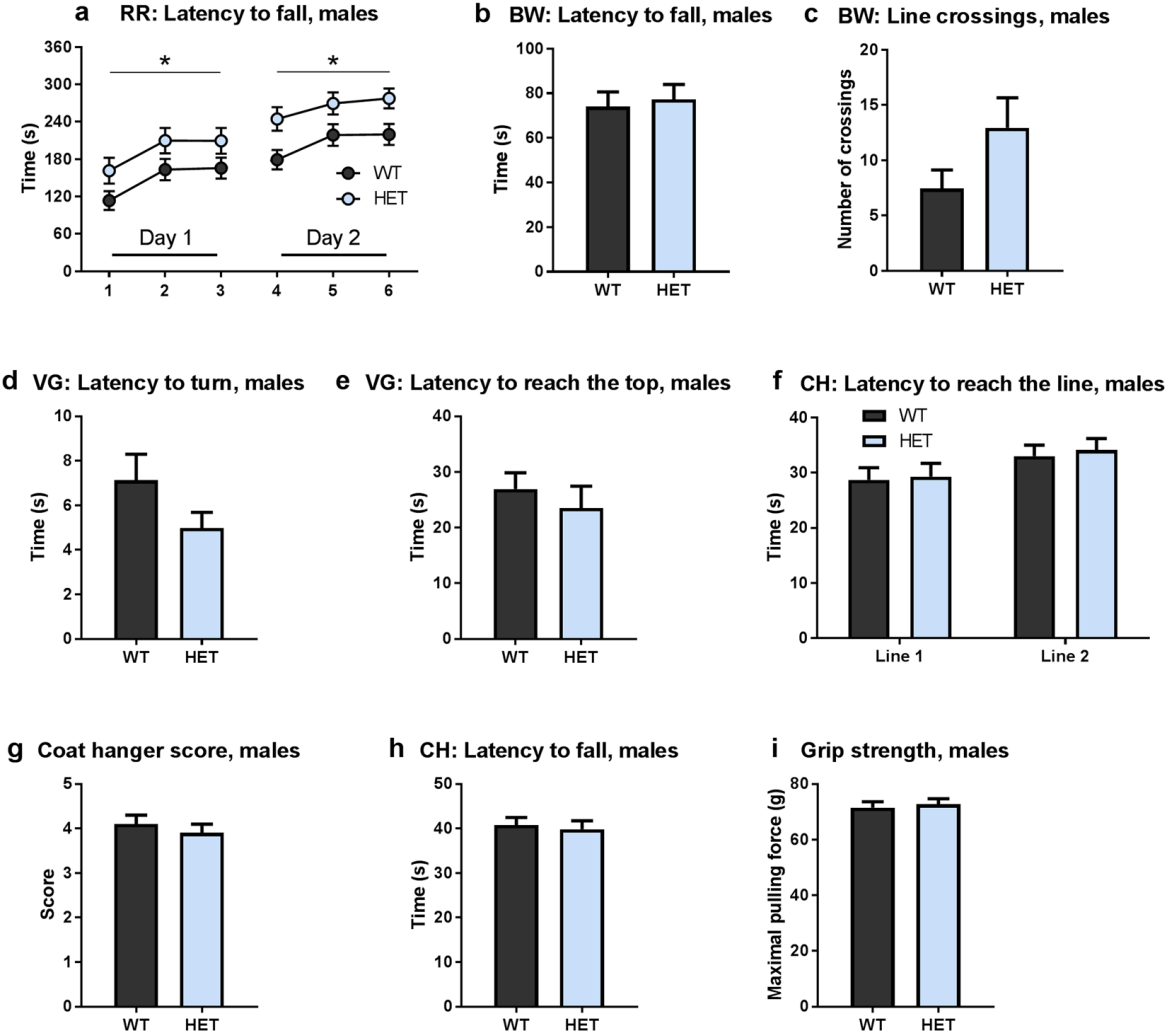
Motor function is improved in *Gdnf* ^*wt/hyper*^ male mice (**a**) Latency to fall off the accelerating rotarod in each trial. Two-way repeated measures ANOVA, genotype effect Day 1 p = 0.046, Day 2 p = 0.011. (**b**) Latency to fall off the beam in the beam walking test. Mann-Whitney U-test, p = 0.63. (**c**) The number of crossed lines in the beam walking test. Student’s t-test with Welch’s correction, p = 0.092. (**d**) Latency to turn in the vertical grid test. Student’s t-test with Welch’s correction, p = 0.13. (**e**) Latency to reach the top of the grid in the vertical grid test. Student’s t-test, p = 0.48. (**f**) Latency to reach the lines in the coat hanger test. Mann-Whitney U-test, Line 1 (end of horizontal part) p = 0.84, Line 2 (on the diagonal part) p = 0.80. (**g**) Coat hanger test score. Mann-Whitney U-test, p = 0.67. (**h**) Latency to fall in the coat hanger test. Mann-Whitney U-test, p = 0.60. (**i**) Maximal forepaw pulling force in the grip strength test. Student’s t-test, p = 0.70. Abbreviations: BW, beam walking; CH, coat hanger; RR, rotarod; VG, vertical grid. (**a**–**c**) N = 34 *Gdnf* ^*wt/wt*^, 31 *Gdnf* ^*wt/hyper*^. (**d**–**h**) N = 24 *Gdnf* ^*wt/wt*^, 21 *Gdnf* ^*wt/hyper*^. (**i**) N = 33 *Gdnf* ^*wt/wt*^, 31 *Gdnf* ^*wt/hyper*^. Data is presented as mean ± SEM. *p < 0.05.

First, we utilized the accelerating rotarod test, which measures motor coordination and balance. The test was performed for 6 minutes, with accelerating speed from 4 to 40 rpm over the first 5 minutes and a constant speed of 40 rpm during the last minute. The test was performed on two consecutive days, with 3 trials performed each day. Both groups learned the task in a similar fashion, over 6 trials exhibiting an increased latency to fall. However, *Gdnf* ^*wt/hyper*^ male mice performed significantly better throughout the trials on each day (Fig. 2a), suggesting that an increase in endogenous GDNF expression improves motor coordination and balance.

Next we used the beam walking test. Mice were placed in the center of a horizontal round beam, and the time to fall off the beam and the number of sections crossed during 2 minutes were recorded. Both genotypes displayed a similar latency to fall off the beam (Fig. 2b). However, *Gdnf* ^*wt/hyper*^ males exhibited a tendency to cross more lines while walking on the beam, compared to *Gdnf* ^*wt/wt*^ mice (Fig. 2c), possibly related to enhanced motor abilities and balance control when moving in challenging conditions. Notably, females were only tested in the first cohort and no differences between genotypes were found in either the rotarod or the beam walking test (Suppl Fig. 1a–c).

Due to the observed difference in motor function in Cohort 1, we performed three additional motor tasks evaluating motor coordination in Cohorts 2 and 3. In the vertical grid test, each animal was placed on a horizontal wire grid, which was raised immediately to a vertical position with the mouse facing the floor at the lower edge. Mice were followed for up to 1 minute and the time to turn upward, to climb to the upper edge or to fall off from the grid were recorded. We found that *Gdnf* ^*wt/hyper*^ mice showed a tendency to turn upward faster than the *Gdnf* ^*wt/wt*^ mice, although there was no statistically significant difference (Fig. 2d). Also, the time to reach the upper edge of the grid was similar between genotypes (Fig. 2e).

Next, behavior was assessed using the coat hanger test. The animals were placed to hang at the center of a horizontal bar with forepaws. The body position of the animal was observed for 45 s and scored on a scale from 0 to 5, with 0 indicating the worst possible outcome (falling off within 10 s) and 5 the best possible performance (active escape to the end of the bar). In addition, the performance was characterized by measuring the time to reach the end of the wire (Line 1) and to obtain upward position (Line 2). There were no differences between genotypes in either the latency to reach the lines, coat hanger score or latency to fall (Fig. 2f–h).

Finally, forelimb grip strength was measured to determine if the improved motor coordination observed in the rotarod test could be attributed to increased muscle tone. We found that the maximal pulling force was similar between *Gdnf* ^*wt/wt*^ and *Gdnf* ^*wt/hyper*^ groups in both males and females (Fig. 2i, Suppl Fig. 1d), indicating that improved performance in the rotarod test is most likely not caused by increased muscle strength.

### Nociception and olfaction

Given that *Gdnf* ^*wt/hyper*^ mice had improved motor coordination compared to controls, we became interested in studying whether an increase in endogenous GDNF expression and/or the concomitant increase in the midbrain dopaminergic system cause changes in any other aspects of behavior.

We started by analyzing nociception and olfaction, since defects in either of these may change the results and interpretation of other behavioral tests. Ectopic GDNF application has been shown to influence the survival and electrophysiological properties of IB4-positive nociceptive neurons in the dorsal root ganglia (DRG)^24,25^. In addition, intrathecal infusion of recombinant human GDNF after unilateral sciatic nerve axotomy protected IB4-positive DRG and spinal cord dorsal horn neurons^25^. Therefore, elevated levels of endogenous GDNF could support the survival of nociceptive neurons and thus increase the sensitivity of animals to mechanical and thermal stimuli^26^.

**Figure 3:**
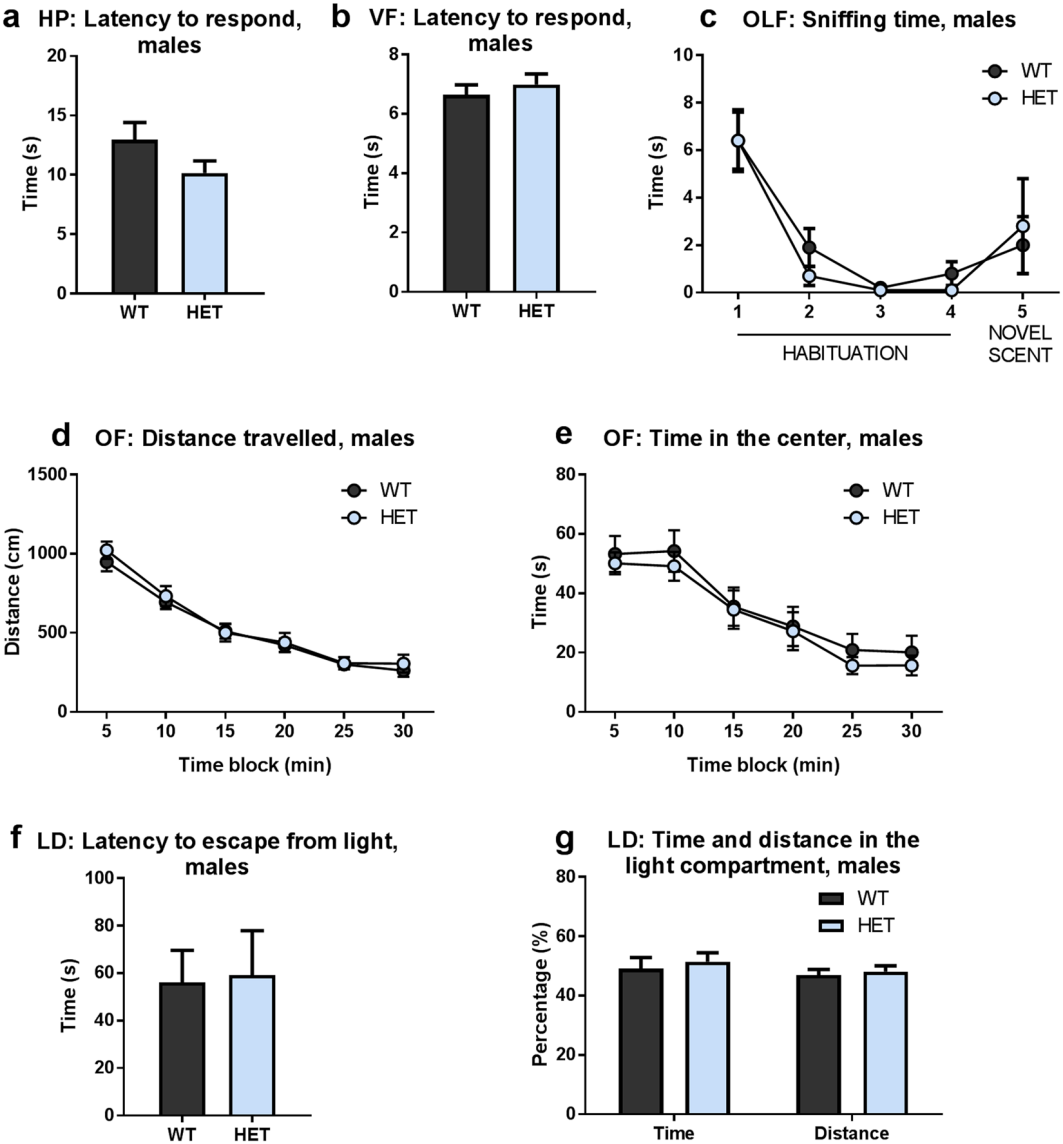
Nociception, olfaction, locomotor activity and anxiety are not altered in *Gdnf* ^*wt/hyper*^ mice (**a**) Latency to respond in the hot plate test; males. Student’s t-test, p = 0.13. (**b**) Latency to respond in von Frey test; males. Student’s t-test, p = 0.49. (**c**) Sniffing time during four trials of habituation to a scent sample and during dishabituation to a novel scent; males. Two-way ANOVA, genotype effect p = 0.74. (**d**) Distance travelled in the open field in 5-minute blocks; males. Two-way repeated measures ANOVA, genotype effect p = 0.58. (**e**) Time spent in the center of the open field in 5-minute blocks; males. Two-way repeated measures ANOVA, genotype effect p = 0.59. (**f**) Latency to escape from the light compartment in the light-dark test; males. Student’s t-test, p = 0.89. (**g**) Percentage of time spent and distance travelled in the light compartment in the light-dark test; males. Student’s t-test, p = 0.63 (time), p = 0.67 (distance). Abbreviations: HP, hot plate test; LD, light-dark test; OF, open field; OLF, olfactory habituation/dishabituation task; VF, von Frey test. (**a**,**c**,**f**,**g**) N = 10 *Gdnf* ^*wt/wt*^, 10 *Gdnf* ^*wt/hyper*^. (**b**) N = 14 *Gdnf* ^*wt/wt*^, 14 *Gdnf* ^*wt/hyper*^. (**d**,**e**) N = 34 *Gdnf* ^*wt/wt*^, 31 *Gdnf* ^*wt/hyper*^. Data is presented as mean ± SEM.

To analyze nociception in *Gdnf* ^*wt/hyper*^ mice, we used the hot plate and von Frey tests. For thermal stimulation, the mice were placed on a plate heated to 52 °C and the time before licking or shaking a hind paw was recorded. We found no significant difference in the latency to pain reflex between the genotypes (Fig. 3a, Suppl Fig. 2a). To evaluate sensitivity to mechanical stimulation, a small probe was used for applying force on either of the hind paws and the time until paw withdrawal was measured. Similarly to the hot plate test, there was no difference between groups in the von Frey test (Fig. 3b). Therefore, increased endogenous GDNF expression does not increase sensitivity to mechanical or thermal stimuli.

Olfactory function of *Gdnf* ^*wt/hyper*^ was studied for two additional reasons besides the possible behavioral implications. First, *in vitro* experiments propose that GDNF may regulate the migration of neural precursors from the subventricular zone to the olfactory bulb along the rostral migratory stream^27^. Therefore mice with increased endogenous GDNF expression could have changes to the olfactory system that affect the sense of smell. Second, hyposmia (losing the sense of smell) is one of the most common features of PD, presenting in at least 90% of patients^28^. The reasons for this are still unknown but may partly be related to dopamine neuron dysfunction (for review, see^29^). Furthermore, a direct dopaminergic nigro-olfactory projection was recently described, possibly linking olfactory dysfunction and PD progression^30^.

To evaluate gross olfactory function, we used the olfactory habituation/dishabituation test. A sample (cinnamon or cocoa) was placed into a clean cage where the mouse had had time to adapt. The latency to approach and the time spent in sniffing the sample during 1 min trial were measured. Habituation was repeated for 4 times. Dishabituation was induced by placing a sample containing a novel scent in the cage for 1 min. We found that the latency to approach the sample and sniffing time per 1 min trial were similar during habituation and dishabituation between the groups in both males (Fig. 3c) and females (Suppl Fig. 2b), suggesting similar olfactory function. In conclusion, there seems to be no gross olfactory deficits in olfaction in *Gdnf* ^*wt/hyper*^ mice.

### Locomotor activity and anxiety-like behavior

Striatal dopamine levels have been associated with general locomotor activity. For example, a 50% decrease in striatal dopamine levels in mice with reduced MECP2 expression is associated with reduced locomotor activity, impaired motor coordination and motor skill learning^31,32^, while genetic modifications that increase dopamine levels induce hyperlocomotion^33,34^. Similarly, a prolonged elevation in extracellular dopamine levels in DAT knock-out or hypomorphic mice results in hyperactive behavior^12,35–37^, especially in a novel environment^36^.

To assess whether endogenous GDNF overexpression affects spontaneous locomotor activity, exploration or anxiety-like behavior, the animals were analyzed in the open field and light-dark test. First, the animals were released in an open field arena and their activity was recorded for 30 minutes. Overall distance travelled was similar between genotypes in both males (Fig. 3d) and females (Suppl Fig. 2e), suggesting that *Gdnf* ^*wt/hyper*^ mice do not have increased locomotor activity compared to wild-type controls. In addition, there were no differences in the distance travelled, time spent in the center of the arena, or in the number of rearings in either males or females (Fig. 3e, Suppl Fig. 2c–h). These results imply that increased endogenous GDNF expression and the concomitant increase in striatal dopamine levels do not change exploratory behavior in a novel environment or induce anxiety-like behavior.

To further analyze anxiety-like behavior, we used the light-dark test, in which the animals were initially placed into the illuminated compartment. We found that the latency to escape from light, as well as the percentage of time spent and distance travelled in the light compartment were statistically not different between the genotypes in both males (Fig. 3f,g) and females (Suppl Fig. 2i–j). In summary, spontaneous locomotor and explorative activity, or anxiety-like behavior in *Gdnf* ^*wt/hyper*^ mice are not altered.

### Depression-like behavior and response to natural reward

One of the most important functions of the mesolimbic dopamine system is to mediate responses to natural rewards, such as food, sex and social interaction^38–40^. Given the prominence of anhedonia and reduced motivation in depressed patients, it has been proposed that the same pathway may contribute to the pathophysiology of depression^10^. Notably, in PD, apathy and depression are two of the most prominent non-motor symptoms that significantly affect patients’ quality of life^41^.

**Figure 4:**
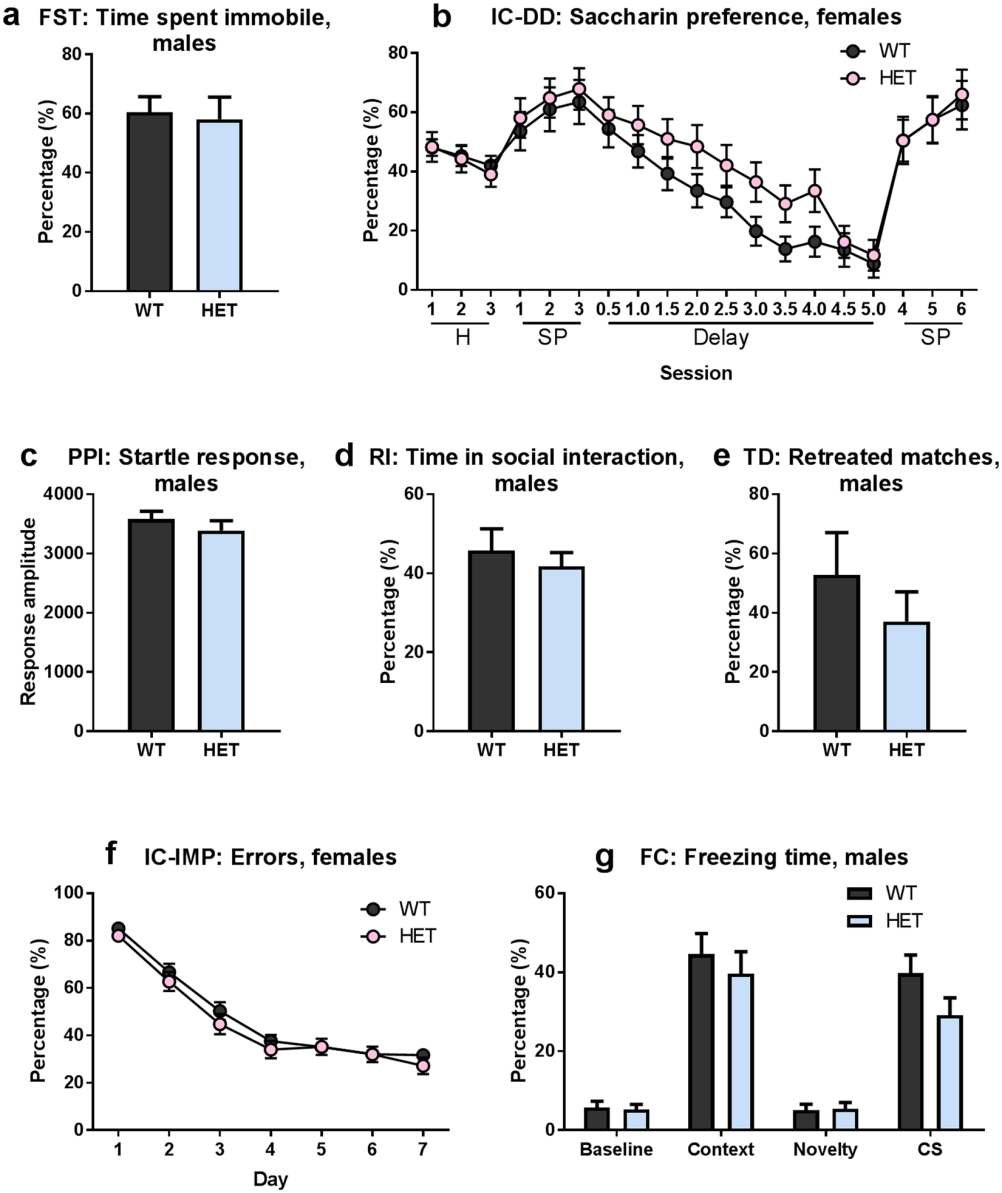
Neither neuropsychiatric disorder-related behaviors nor learning and memory are changed in *Gdnf* ^*wt/hyper*^ mice (**a**) Percentage of time spent immobilized in the forced swim test; males. Student’s t-test, p = 0.81. (**b**) Percentage of licks at corners containing saccharin (saccharin preference) in the IntelliCage delay discounting task; females. Two-way repeated measures ANOVA, genotype effect p = 0.78 (habituation), p = 0.65 (saccharin preference), p = 0.14 (delay), p = 0.91 (preference II). (**c**) Average startle response amplitude over 10 trials; males. Student’s t-test p = 0.36. (**d**) Percentage of time spent in social interaction in the resident-intruder test; males. Student’s t-test, p = 0.54. (**e**) Percentage of lost matches in the tube dominance test; males. Student’s t-test, p = 0.37. (**f**) Percentage of premature responses in the motor impulsivity (IMP) task; females. Two-way repeated measures ANOVA, genotype effect p = 0.35. (**g**) Percentage of time spent freezing in the fear conditioning test; males. Multiple Student’s t-tests with Holm-Sidak correction, p = 0.99 (Baseline), p = 0.71 (Context), p = 0.99 (Novelty), p = 0.15 (Conditioned Stimulus). (**a**,**d**) N = 10 *Gdnf* ^*wt/wt*^, 10 *Gdnf* ^*wt/hyper*^. (**b**) N = 19 *Gdnf* ^*wt/wt*^, 17 *Gdnf* ^*wt/hyper*^. (**c**) N = 34 *Gdnf* ^*wt/wt*^, 31 *Gdnf* ^*wt/hyper*^. (**e**) N = 8 *Gdnf* ^*wt/wt*^, 10 *Gdnf* ^*wt/hyper*^. (**f**) N = 20 *Gdnf* ^*wt/wt*^, 19 *Gdnf* ^*wt/hyper*^. (**g**) N = 32 *Gdnf* ^*wt/wt*^, 30 *Gdnf* ^*wt/hyper*^. Abbreviations: FC, fear conditioning; FST, forced swimming test; H, habituation; IC-DD, delay discounting task; IC-IMP, motor impulsivity task; PPI, prepulse inhibition; RI, resident-intruder test; SP, saccharin preference; TD, tube dominance test. Data is presented as mean ± SEM.

Since *Gdnf* ^*wt/hyper*^ mice have slightly increased dopamine levels in the ventral tegmental area compared to wild-type littermates^42^, we were interested to know if this causes changes in tests measuring depression-like behavior and response to natural reward. Depression-like behavior was analyzed using the forced swimming test, in which we observed no differences in time of immobility between the genotypes in either males or females (Fig. 4a, Suppl Fig. 3a).

To assess motivational behavior, we used the delay discounting paradigm in the IntelliCage. In this task, one bottle of water in each corner was replaced with 0.5% saccharin and the animals were allowed to develop spontaneous preference for saccharin over 3 days (training period). During the next phase (discounting), delays for opening the saccharin doors were gradually increased, whereas door to water opened without the delay. After 10 days, the delay for opening doors leading to saccharin was 5 s. In the last phase of 3 days (extinction), delays were reset to 0 s. We found that both groups showed a similar preference for saccharin and that was gradually reduced over the discounting period when a delay was applied for getting access to sweet solution (Fig. 4b). Therefore, *Gdnf* ^*wt/hyper*^ mice do not develop increased or reduced susceptibility to addiction to natural rewards. Overall our results suggest that increased endogenous GDNF expression has no gross impact on motivational behavior.

### Sensorimotor gating

Altered dopamine system function has been suggested as a potential underlying mechanism of schizophrenia. Several studies have reported that patients with schizophrenia have elevated presynaptic striatal dopamine synthesis capacity and increased dopamine release (reviewed in^11^). Schizophrenia is difficult to model in mice, as most of the associated symptoms cannot be identified from mouse behavior^43^. Therefore, the endophenotype concept is recommended for modeling complex disorders^44^. As of now the only human schizophrenia endophenotype that is reliably reproducible in mice is reduced prepulse inhibition^45,46^. In the prepulse inhibition test a mouse is subjected to a startle stimulus (SS) preceded by a weaker pre-pulse stimulus (PPS). In healthy people and mice the response to the SS is reduced if it is preceded by the PPS. In contrast, schizophrenic patients and mice with schizophrenia-like phenotype react to the SS with a similar response amplitude, regardless of whether the PPS was presented or not^47,48^.

We tested prepulse inhibition in *Gdnf* ^*wt/wt*^ and *Gdnf* ^*wt/hyper*^ mice and found that the average acoustic startle response was similar between genotypes in both males (Fig. 4c) and females (Suppl Fig. 3c). In addition, there was no difference in the extent of prepulse inhibition with prepulse stimuli of different strength Suppl Fig. 3b, d). Therefore, a mild elevation in tissue GDNF and dopamine levels, an increase in evoked dopamine release and increased DAT activity do not cause a schizophrenia-like phenotype in *Gdnf* ^*wt/hyper*^ mice.

### Social behavior

As mentioned above, the mesolimbic dopamine system is highly involved in the behavioral response to reward anticipation and to prediction errors^40^. Aggressive behavior often arises when an individual has to compete for rewards or if the expectation of a reward is challenged by others. Accordingly, several studies have associated aggressive behavior with dopamine signaling^49–53^. Along the same line, changes in mood and personality, including increased aggressive behavior, have been observed in some Parkinsonian patients^54^.

Aggression-related social behavior was evaluated with two tests: the resident-intruder test and tube dominance test. In the resident-intruder test, an intruder mouse was put into the cage where the test mouse had been habituating for 30 minutes. We found that *Gdnf* ^*wt/wt*^ and *Gdnf* ^*wt/hyper*^ male mice spent comparable time in social interaction (Fig. 4d).

In the tube dominance test, two mice of the same sex but of different genotypes were placed in the opposite ends of a plastic tube and released simultaneously. The match ended when one mouse completely retreated from the tube. We observed no statistically significant differences in the percentage of retreated matches between the genotypes in either males or females (Fig. 4e). Taken together, the results suggest that social behavior is not affected by enhanced levels of GDNF in mice.

### Impulsivity

Attention-deficit hyperactivity disorder (ADHD) is characterized by three core features: hyperactivity, impulsivity and difficulties in sustaining attention^55,56^. Dopamine system dysfunction is considered one of the main mechanisms in ADHD pathophysiology and genes involved in the dopamine pathway, especially the dopamine transporter, have been associated with the disease^12,13^.

The features of ADHD can present in various ways in both adults and children. One of the characteristic behaviors related to impulsivity is the inability to wait for one’s turn. To see potential changes in impulsivity, we tested the *Gdnf* ^*wt/hyper*^ mice in the IntelliCage motor impulsivity task, which requires the mice to learn to wait for an appropriate, randomly-applied time to have access to water following a first nose-poke^57^. We found that *Gdnf* ^*wt/wt*^ and *Gdnf* ^*wt/hyper*^ mice performed similarly in this task (Fig. 4f, Suppl Fig. 3f). Furthermore, as described earlier, we found that *Gdnf* ^*wt/hyper*^ mice do not exhibit increased motor activity in the open field test compared to wild-type mice (Fig. 3d,e, Suppl Fig. 2c–h). Those results suggest that a 2-fold increase in endogenous GDNF expression does not induce an ADHD-like phenotype.

### Learning and memory

*Gdnf* ^*wt/KO*^ mice expressing reduced levels of endogenous GDNF have deficits in the water maze task^14^, and overexpression of ectopic GDNF in the hippocampus improves spatial learning in rats^58^, suggesting that GDNF may be involved in neuronal plasticity and cognitive function. We tested learning and memory in *Gdnf* ^*wt/hyper*^ mice using the fear conditioning test and different tasks in the IntelliCage. Both genotypes displayed similarly-enhanced freezing in testing contextual and cued memory after classical fear conditioning (Fig. 4g, Suppl Fig. 3e). In the IntelliCage, mice were presented increasingly challenging learning and memory tasks known to be sensitive in animal models of impaired memory^59,60^. We found no differences in the percentage of incorrect visits with nose-pokes in four experimental designs assessing learning and memory: the corner preference (CP), serial reversal (SR), chaining (CHAIN) and patrolling (PATR) tasks (Suppl Fig. 3g–i), indicating that both groups were able to learn the tasks equally. Therefore, an increase in endogenous GDNF expression does not induce gross changes in learning and memory.

### Summary

We have analyzed the behavioral effects of increased endogenous GDNF expression in *Gdnf* ^*wt/hyper*^ mice^9^ with 20 different behavioral tests. We found that motor coordination and balance are improved in *Gdnf* ^*wt/hyper*^ mice. GDNF is expressed in multiple sites involved in motor control (e.g., striatum, cerebellum, spinal cord and muscles), and future studies will demonstrate, which of the above are responsible for the improved motor function.

*Gdnf* ^*wt/hyper*^ mice have a modest (~20%) increase in tissue dopamine levels in the dorsal striatum, substantia nigra and ventral tegmental area, compared to wild-type controls^9,42^. This mild increase in tissue dopamine levels is accompanied by increase in evoked dopamine release and by a 5-fold increase in DAT activity in the dorsal striatum^9^. Changes in dopamine system function, including increased or decreased dopamine levels and/or altered DAT activity, have been associated with a variety of neuropsychiatric diseases such as schizophrenia^11^, ADHD^13^ and depression^10^. Furthermore, dopamine neuron degeneration in PD is thought to contribute to the emergence of several PD-associated non-motor symptoms such as cognitive problems, sleep disorders, hyposmia, altered mood and social difficulties^54^. Therefore it was of importance to study the behavioral consequences of both increased endogenous GDNF expression and changes in the dopamine system of *Gdnf* ^*wt/hyper*^ mice.

Animal studies on Parkinson’s disease models and clinical trials have reported variable outcomes of GDNF overexpression via protein infusion or viral transduction^61^. The expression levels of ectopic GDNF achieved in these studies usually exceed endogenous GDNF levels by up to hundreds or thousands of times^4,9,62,63^, causing potentially undesirable effects on the dopamine system, including massive sprouting of dopamine neuron fibers towards the site of GDNF delivery^3,4,64^ and downregulation of tyrosine hydroxylase levels in the striatum^4,62,63,65^. In *Gdnf* ^*wt/hyper*^ mice we did not observe changes in striatal TH expression, and dopamine fiber density was comparable to wild-type mice^9^, suggesting that the observed outcomes after ectopic GDNF delivery most likely occur due to abnormally high GDNF levels. In line with this hypothesis, another study recently investigated the outcome of GDNF overexpression from a doxycycline-inducible viral vector on dopamine system function. Using different doses of doxycycline, the authors achieved high (12-fold) and low (3-fold) GDNF overexpression, and showed that only the former caused significant changes in TH expression^66^. The study also reported that both high and low levels of ectopic GDNF decreased dopamine transporter activity^66^. At the same time, studies addressing the role of endogenous—rather than ectopic—GDNF have shown that both a ~50% reduction and 2-fold increase in endogenous GDNF levels increase dopamine transporter activity^9,67,68^, altogether suggesting that dopamine system function is sensitive both to the levels and source of GDNF.

Other adverse effects observed in animals after nigrostriatal delivery of recombinant protein or viral vector overexpressing GDNF are hyperactivity and reduced body weight^69–74^. However, neither of these phenotypes were observed in *Gdnf* ^*wt/hyper*^ mice^9^, again implying that the site and extent of GDNF overexpression is important. However, using currently available methods it is difficult to estimate how ectopic GDNF expression pattern and GDNF levels influence the outcome of clinical trials.

Our results suggest that about 2-fold increase in endogenous GDNF expression does not induce behavioral changes commonly associated with dopamine system dysfunction or ectopic GDNF overexpression. *Gdnf* ^*wt/hyper*^ mice did not exhibit changes in tests measuring nociception, olfaction, locomotor activity, aggression, response to natural reward, or learning and memory; nor did they develop gross ADHD-, anxiety-, depression- or schizophrenia-like phenotype. Overall we conclude that 2-fold increase in endogenous GDNF expression specifically improves motor coordination without affecting other main central nervous system functions.

## Electronic supplementary material

Supplementary Information

Supplementary Table 1
