# Supplementary material for: Two-fold elevation of endogenous GDNF levels in mice improves motor coordination without causing side-effects
Source: Sci Rep. 2018 Aug 8;8:11861. doi: 10.1038/s41598-018-29988-1 (PMC6082872; doi:10.1038/s41598-018-29988-1)
Supplement: Supplementary file 1 — Supplementary Information [file 41598_2018_29988_MOESM1_ESM.pdf]

Title: Two-fold elevation of endogenous GDNF levels in mice improves motor coordination without causing side-effects

Authors: Kärt Mätlik[1]*, Vootele Võikar*, Carolina Vilenius[1], Natalia Kulesskaja, Jaan-Olle Andressoo[1]

Supplementary Figure legends

Supplementary Figure 1 Motor function in Gdnf$^{wt/hyper}$ females is not changed a Latency to fall off the accelerating rotarod in each trial. Two-way repeated measures ANOVA, genotype effect Day 1 p = 0.67, Day 2 p = 0.25. b Latency to fall off the beam in beam walking test. Mann-Whitney U-test, p = 0.85. c The number of crossed lines in the beam walking test. Mann-Whitney U-test, p = 0.62. d Maximal forepaw pulling force in the grip strength test. Student's t-test, p = 0.66. Abbreviations: BW, beam walking; RR, rotarod. a-d N = 10 Gdnf$^{wt/wt}$, 9 Gdnf$^{wt/hyper}$. Data is presented as mean ± SEM.

Supplementary Figure 2 Pain, olfaction, locomotor activity and anxiety are not changed in Gdnf$^{wt/hyper}$ mice a Latency to respond in the hot plate test; females. Student's t-test, p = 0.54. b Sniffing time during four trials of habituation to a scent sample and during dishabituation to a novel scent; females. Two-way ANOVA, genotype effect p = 0.62. c Percentage of distance travelled in the center of the open field in 5-minute blocks; males. Two-way repeated measures ANOVA, genotype effect p = 0.68. d Number of rearings in the open field in 5-minute blocks; males. Two-way repeated measures ANOVA, genotype effect p = 0.51. e Distance travelled in the open field in 5-minute blocks; females. Two-way repeated measures ANOVA, genotype effect p = 0.48. f Percentage of distance travelled in the center of the open field in 5-minute blocks; females. Two-way repeated measures ANOVA, genotype effect p = 0.16. g Time spent in the center of the open field in 5-minute blocks; females. Two-way repeated measures ANOVA, genotype effect p = 0.25. h Number of rearings in the open field in 5-minute blocks; females. Two-way repeated measures ANOVA, genotype effect p = 0.54. i Latency to escape from the light compartment in the light-dark test; females. Student's t-test with Welch's correction, p = 0.082. j Percentage of time spent and distance travelled in the light compartment in the light-dark test; females. Student's t-test, p = 0.057 (time), p = 0.13 (distance). Abbreviations: LD, light-dark test; OF, open field; OLF, olfactory habituation/dishabituation task. a N = 10 Gdnf$^{wt/wt}$, 8 Gdnf$^{wt/hyper}$. b 9 Gdnf$^{wt/wt}$, 9 Gdnf$^{wt/hyper}$. c-d N = 34 Gdnf$^{wt/wt}$, 31 Gdnf$^{wt/hyper}$. e-j N = 10 Gdnf$^{wt/wt}$, 9 Gdnf$^{wt/hyper}$. Data is presented as mean ± SEM.

Supplementary Figure 3 Neuropsychiatric disorder-related behaviors are not changed in Gdnf$^{wt/hyper}$ mice a Percentage of time spent immobilized in the forced swim test; females. Student's t-test with Welch's correction, p = 0.34. b Response to startle stimulus with different prepulse stimuli, relative to response to startle stimulus alone; males. Two-way repeated measures ANOVA, genotype effect p = 0.85. c Average startle response amplitude over 10 trials; females. Student's t-test p = 0.84. d Response to startle stimulus with different prepulse stimuli, relative to response to startle stimulus alone; females. Two-way repeated measures ANOVA, genotype effect p = 0.82. e Percentage of time spent freezing in the fear conditioning test; females. Multiple Student's t-tests with Holm-Sidak correction, p = 0.79 (Baseline), p = 0.79 (Context), p = 0.79 (Novelty), p = 0.34 (Conditioned Stimulus). f Total number of corner visits in the motor impulsivity (IMP) task; females. Two-way repeated measures ANOVA, genotype effect p = 0.29. g Percentage of incorrect corner visits in the corner preference—serial reversal (CP-SR) task; females. Two-way repeated measures ANOVA, genotype effect p = 0.10 (corner preference), p = 0.11 (serial reversal). h Percentage of incorrect corner visits in the patrolling (PATR) task; females. Two-way repeated measures ANOVA,

genotype effect $p = 0.35$. i Percentage of incorrect corner visits in the chaining (CHAIN) task; females. Two-way repeated measures ANOVA, genotype effect $p = 0.35$. Abbreviations: CP, corner preference; FC, fear conditioning; FST, forced swimming test; H, habituation; IC-CHAIN, chaining task; IC-CP-SR, corner preference—serial reversal task; IC-DD, delay discounting task; IC-IMP, motor impulsivity task; IC-PATR, patrolling task; PPI, prepulse inhibition; SP, saccharin preference; SR, serial reversal. a N = 9 $Gdnf^{wt/wt}$, 9 $Gdnf^{wt/hyper}$. b N = 34 $Gdnf^{wt/wt}$, 31 $Gdnf^{wt/hyper}$. c-e N = 10 $Gdnf^{wt/wt}$, 9 $Gdnf^{wt/hyper}$. f-i N = 21 $Gdnf^{wt/wt}$, 19 $Gdnf^{wt/hyper}$. Data is presented as mean ± SEM.

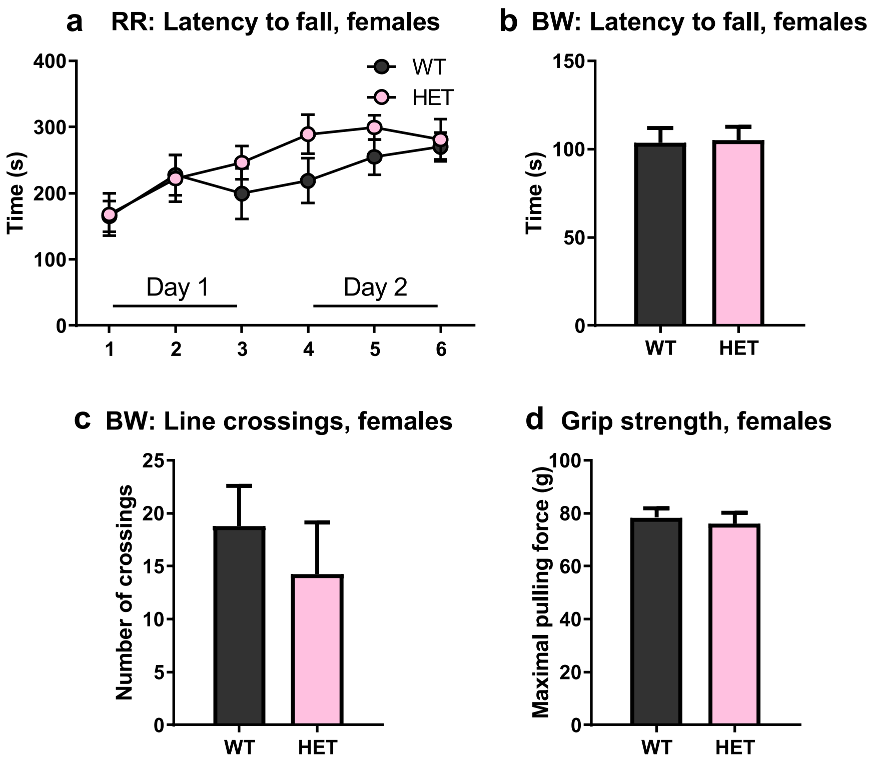

a
RR: Latency to fall, females
400
300
200
100
0
Time (s)
WT
HET
Day 1
Day 2
1
2
3
4
5
6
b
BW: Latency to fall, females
150
100
50
0
Time (s)
WT
HET
c
BW: Line crossings, females
25
20
15
10
5
0
Number of crossings
WT
HET
d
Grip strength, females
100
80
60
40
20
0
Maximal pulling force (g)
WT
HET

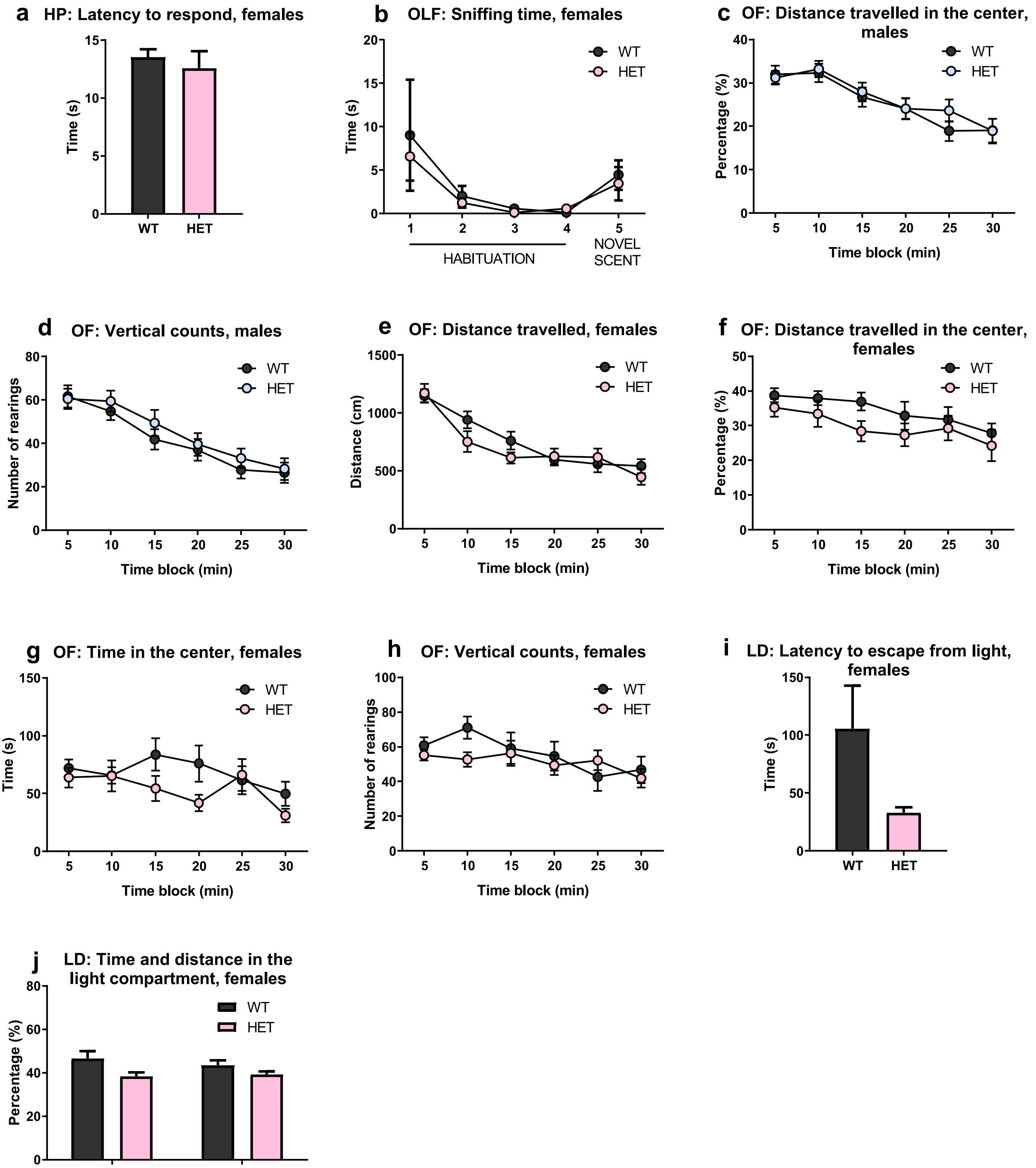
a HP: Latency to respond, females
Time (s)
WT
HET
b OLF: Sniffing time, females
Time (s)
WT
HET
HABITUATION
NOVEL SCENT
c OF: Distance travelled in the center, males
Percentage (%)
Time block (min)
WT
HET
d OF: Vertical counts, males
Number of rearings
Time block (min)
WT
HET
e OF: Distance travelled, females
Distance (cm)
Time block (min)
WT
HET
f OF: Distance travelled in the center, females
Percentage (%)
Time block (min)
WT
HET
g OF: Time in the center, females
Time (s)
Time block (min)
WT
HET
h OF: Vertical counts, females
Number of rearings
Time block (min)
WT
HET
i LD: Latency to escape from light, females
Time (s)
WT
HET
j LD: Time and distance in the light compartment, females
Percentage (%)
WT
HET
Time
Distance

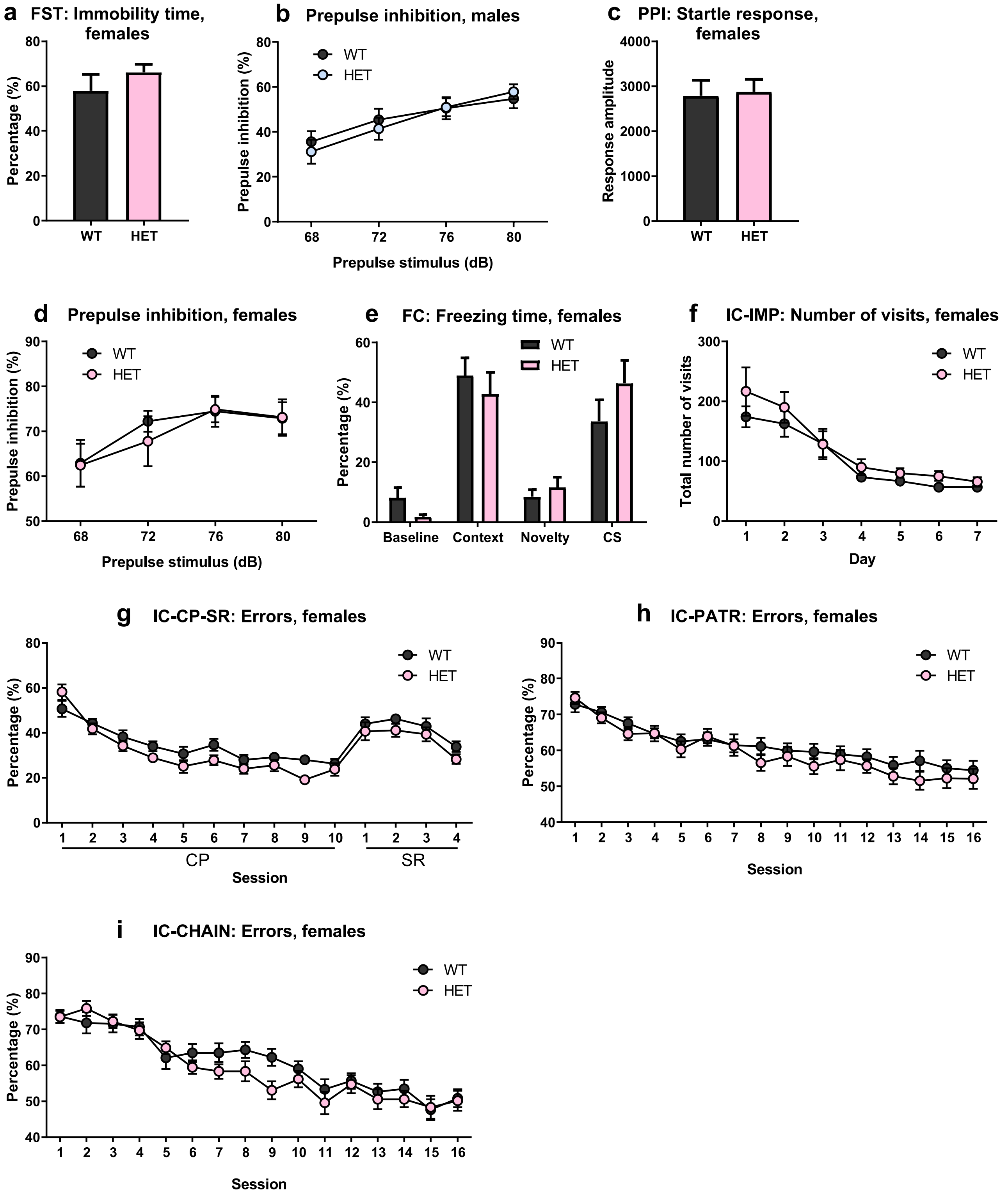

a FST: Immobility time, females
Percentage (%)
WT
HET
b Prepulse inhibition, males
Prepulse inhibition (%)
Prepulse stimulus (dB)
WT
HET
c PPI: Startle response, females
Response amplitude
WT
HET
d Prepulse inhibition, females
Prepulse inhibition (%)
Prepulse stimulus (dB)
WT
HET
e FC: Freezing time, females
Percentage (%)
Baseline
Context
Novelty
CS
WT
HET
f IC-IMP: Number of visits, females
Total number of visits
Day
WT
HET
g IC-CP-SR: Errors, females
Percentage (%)
CP
SR
Session
WT
HET
h IC-PATR: Errors, females
Percentage (%)
Session
WT
HET
i IC-CHAIN: Errors, females
Percentage (%)
Session
WT
HET
